# Supplementary material for: Diversity in approaches in community-based mental health interventions in India: A narrative review and synthesis
Source: Glob Ment Health (Camb). 2025 Jul 30;12:e89. doi: 10.1017/gmh.2025.10046 (PMC12394029; doi:10.1017/gmh.2025.10046)
Supplement: Gundi et al. supplementary material [file S2054425125100460sup001.docx]

**S1 Appendix: Intervention Components and Details of the Approaches in the selected CMH interventions in India**

| **S No** | **Intervention Name**  **and**  **primary implementing institution** | **Population in Focus** | **Approaches and Implementation Platforms** | | | | | |
| --- | --- | --- | --- | --- | --- | --- | --- | --- |
|  |  |  | **1. Primary Prevention and Health Promotion at community platforms** | **2. Identification and case detection at community platforms** | **3. Identification and case detection at health care platforms** | **4. First line of mental health care at community platforms** | **5. Treatment and care at healthcare platforms** | **6. Rehabilitation at community platforms** |
| i1 | **Asia Psychosocial Rehabilitation Programme**  *Dr. Somerville Memorial CSI Medical College and Hospital* | People with mental illness (PwMI), women in local communities | 1. Community volunteers conduct promotion and prevention activities through discussions with children, adolescents and adults on topics such as: stress management, study skills, substance use, mental illness, nutrition, hygiene, communicable and chronic illnesses   2. Public meetings to provide information about services offered | 1. Community volunteers engaged in active case finding through home visits and consulting key informants  2. Schoolteachers trained to identify and refer children with behavioural problems  3. Diagnosis at camps by specialist | | 1. Community volunteers conduct follow-up visits where they provide informal counselling (through active listening and relaxation techniques), deliver medicines at home, and provide support to caregivers   2. Monthly psychiatric community clinics in two areas involving social workers and psychiatrist  3. Medication provided free-of-charge   4. Psychotherapy provided by a psychotherapist. For one area, patients travel to the hospital out-patient department for psychiatric treatment   5. Inpatient care for people with acute psychiatric episodes | | 1. Community volunteers help patients with self-care, activities of daily living (ADL) and social functioning during follow up sessions   2. Self-Help Groups- open to all women- provide income-generation activities and opportunities for social integration to PwMI |
| i2 | **Atmiyata**  *Centre for Mental Health Law and Policy -CMHLP* | People with mental distress or mental illness, local community members | 1. Champions screen films on social determinants of mental health (e.g. domestic violence and substance use) with small groups of community members | 1. Champions identify and provide psychosocial support   2. Mitras identify people with distress or mental illness and refer them to champions |  | 1. Six counselling sessions through techniques like active listening, behavioral activation, problem solving and social support, conducted by Champions, under the supervision of facilitators who are social workers   2. Follow-ups done over the phone or in-person by Champions |  | 1. Champions facilitate the process of applying for and attaining social benefits for people with common mental disorders and severe mental disorders |
| i3 | **BasicNeeds Model of Mental Health and Development**  *BasicNeeds India (in collaboration with partner NGOs)* | PwMI | 1. Awareness programs with caregivers, traditional healers, local health personnel and community leaders focused on de-stigmatizing mental illness | 1. Lay health workers identify PwMI  2. Diagnosis at camps by specialists | | 1. Mental health camps - with mental health professionals providing treatment  2. Mental health professionals visit PHCs or community centers to provide treatment and care  3. Follow-up through home visits by community health workers | | 1. Livelihood support for PwMI through mentoring, providing information on livelihood opportunities and liaising with local institutions that can provide employment    2. Encouraging the creation of self-help groups, who provide peer support to manage mental illness and share information to raise awareness in communities |
| i4 | **Community Based Rehabilitation (CBR), Jagaluru**  *National Institute of Mental Health and Neurosciences (NIMHANS) and Association for People with Disability (NGO partner)* | PwMI |  | 1. Accredited Social Health Activists (ASHAs) identified and referred patients to mental health camps | 1. Diagnosis at PHCs and Taluk (block-level) hospitals | 1. Follow-up visits by social workers and ASHAs, where they supervise treatment and provide psychoeducation to family members   2. When patients are unable to visit the clinic, a psychiatrist visits them at home | 1. Mental health camps conducted twice a month at PHCs- psychiatrist provides treatment and prescribes medicine   2. Mental health camps are conducted by psychiatrists twice a month at taluk (block-level) hospitals | 1. Facilitating disability certification and welfare benefits (e.g. disability pension, health insurance, job card) by social workers  2. Caregivers are educated on rights of PwMI through residential camps |
| i5 | **Community Care for People with Schizophrenia in India (COPSI)**  *Sangath, Schizophrenia Research Foundation (SCARF), Chennai*  *Parivartan and Nirmittee (in collaboration with academic institutions)* | People with schizophrenia |  | 1. Key informant community survey to identify participants in one location | 1. Participants recruited from psychiatric clinics- in two locations- screening done by psychiatrists | 1. Primary form of intervention delivery was through home visits by community health workers, who would provide psychoeducation to participants and caregivers, establish adherence management strategies, and help with health promotion strategies for physical health | 1. Facility-based treatment at private clinics and in-patient facilities | 1. Individualized rehabilitation strategies to address personal, social and work areas    2. Linkage to Self-Help groups and methods of user-led support by community health workers   3. Community health workers network with community agencies to enable participants to access employment opportunities, address stigma and help with facilitating legal benefits  4. Pre-vocational and vocational training at home and through referrals to external training programs. |
| i6 | **Community Interventions in Psychotic Disorders (CoInPsyD)**  *National Institute of Mental Health and Neurosciences (NIMHANS)* | People with Severe Mental Disorders (SMDs) |  | 1. Village health workers identify people with severe mental disorders in the community and refer them to the project team   2. Social workers interviewed key informants such as health workers and members of local government to identify people with SMDs | 1. Confirmatory diagnosis by specialists | 1. During enrolment, social workers provide counselling and psychoeducation    2. Village health workers and social workers coordinate treatment and conduct follow up home visits   3. Treatment coordination by social workers from the local community who scheduled follow-ups | 1. At PHCs, psychiatrist prescribes medicines and gives psychoeducation to family members |  |
| i7 | **Community mental health programme**  *Ashadeep* | PwMI and disability | 1. Mental health awareness programs with local government officials, police, teachers and students focused on reducing stigma, dispelling myths and emphasizing the need for treatment | 1. Community members refer cases to the program team | 1. Diagnosis done at outpatient clinics | 1. Home-based psychoeducation by community health worker | 1. Mental health camps and psychiatric OPD held at PHCs, CHCs and in partnership with local NGOs |  |
| i8 | **Community Mental Health Programme (CMHP)**  *Association for Health Welfare in the Nilgiris (ASHWINI)* | PwMI from tribal communities (which are members of a local tribal collective called Adivasi Munnetra Sangam) | 1. Incorporating mental health awareness into existing health education program conducted by Health Animators and Village Health Guides: using street plays, skits, posters, flip charts and videos | 1. Village Health Guides identified PwMI | 1. Diagnosis is done at the area centers or at the hospital by general doctors | 1. Village Health Guides provide basic care and first aid at the village level   2. Follow-up care by Village Health Guides and Health Animators- they conduct home visits and log clients' health status on colour-coded cards  3. In some cases, primary care doctors visit patients in the village and prescribe medicines  4. Village Health Guides, Health Animators and doctors motivate patients to seek care, continue treatment and return to work | 1. Treatment provided at Area Health Centers where primary doctors visit at regular intervals   2. Periodic visits by psychiatrists to support primary care doctors   3. Health Animators managed area centers and were also part of providing care   4. Outpatient and inpatient services at Gudalur Adivasi Hospital (GAH) by generalist doctors and nurses for more advanced care  5. Low-cost insurance scheme to support in-patient costs  6. Counselling services and Alcoholics Anonymous meetings at GAH | 1. Health Animators and Village Health Guides motivate clients to return to work and liaise with previous employers   2. Providing animals or hens for livelihood support |
| i9 | **Community Mental Health Programme (CMHP)**  *MINDS Foundation* | PwMI | 1. Community health workers, social workers and mental health professionals organized mental health education workshops: activities include movie screening and pamphlet distribution | 1. Identification of people in need of mental health care and treatment |  | 1. Community health workers assist people to adhere to treatment plans | 1. Free-of-charge transport is provided for PwMI and families to avail psychiatric treatment | 1. Social and vocational rehabilitation programs   2. Peer support groups organized by community health workers |
| i10 | **Community Mental Health Programme (CMHP)**  *Mental Health Action Trust (MHAT)* | People with SMDs, PwMI facing extreme poverty |  | 1. Community volunteers identify potential clients and refer them to the program team | 1. Psychiatrist provides initial diagnosis | 1. Follow up through weekly home visit done by community volunteers, who monitor clients' progress and provide support for daily activities | 1. Psychiatrist prescribes medication (and consults on difficult cases)  2. Weekly outpatient clinics managed by non-medical professionals     3. Medication dispensed at clinic pharmacy   4. Psychotherapy and counselling provided by psychologist and social worker | 1. Rehabilitation daycare center where clients are supported with activities for daily living and behavioral training - this is run by volunteers   2. Food kit and festival kit provided to clients by volunteers |
| i11 | **Community outreach programme**  *Ranchi Institute of Neuropsychiatry and Allied Sciences (RINPAS)* | PwMI | 1. Door-to-door visits and meetings with village leaders to reduce resistance to psychiatric treatment | 1. Identification of patients by health workers from local NGOs    2. Psychiatrist provides diagnosis at camps | | 1. Monthly mental health camps in the community, where a psychiatrist makes a diagnosis and recommends treatment. Counselling is provided by clinical psychologist or psychiatric social worker, nurses and physicians provide support.  2. Local health workers from NGOs conduct follow-ups where they monitor patients and provide psychoeducation   3. Medicines for 1 month provided free-of-cost to patients.  4. Serious cases are taken up at RINPAS hospital | |  |
| i12 | **Dance Movement Therapy for Trauma Recovery**  *Kolkata Sanved* | Survivors of sexual violence and trafficking |  |  |  | 1. Dance Movement Therapy (DMT) sessions for trauma recovery- need assessment, implementation plan and then implementation of DMT sessions done by DMT practitioners, many of whom are from the community  2.Sessions take place in spaces provided by partner organizations (community centers or shelter homes for survivors) |  | 1. Trauma survivors trained as DMT practitioners, some of whom are employed by Kolkata Sanved |
| i13 | **Dava Dua**  *The Altruist* | PwMI coming to Mira Datar dargah for care | 1. Sensitization and training provided to faith-based healers on mental health, identifying symptoms of mental illness, and referral strategies  2. Awareness programs with local community members at places like Anganwadis and government schools   3. Empowering dargah management to improve hygiene and sanitation in dargah (where PwMI regularly stay) | 1. Faith-based healers at the dargah identify and refer PwMI to allopathic practitioners | 1. Psychiatrist provides diagnosis at outpatient clinic |  | 1. Outpatient clinic where psychiatrist prescribes medication and social worker, or psychologist provides basic counselling and psychoeducation   2. Medicines are provided free-of-charge |  |
| i14 | **Depression in Late Life (DIL)**  *Sangath* | People aged 60 and above at risk of anxiety and depression |  | 1. Research assistants visited houses to interview and recruit participants | 1. Identification of participants was done through interviewing people aged 60 and above at PHCs and sub -centers by research assistants | 1. Problem solving therapies delivered by LHWs, focusing on simple behavioural activation, improving active coping, self-efficacy and education on self- management of common medical illnesses |  | 1. LHWs facilitated access to government social schemes for financial assistance |
| i15 | **District Mental Health Programme (DMHP), Karnataka**  *National Mental Health Programme, State Department of Health and Family Welfare, Karnataka* | PwMI, local community members | 1. Information, Education and Communication (IEC) on mental health awareness and stigma reduction at schools and colleges and with stakeholders such as police, judiciary, elected representatives, faith healers and government staff   2. Life skills education   3. Workplace stress management   4. Suicide prevention services | 1 ASHAs and Auxiliary Nurse Midwives (ANMs) identified psychiatric cases and referred them to PHCs where DMHP was being implemented | 1. Psychiatrist provides diagnosis | 1. In some districts, care at doorsteps for people with SMDs who were not regularly visiting DMHP clinics  2. This was done by social worker and DMHP nurse who enquire about reason for dropout, provide psychosocial services and supply medicines | 1. Clinical services at PHCs through psychiatrist  2. Clinical services at CHCs and taluk (block-level) hospital through psychiatrist | 1. Daycare rehabilitation facility – including vocational training, recreation and physical exercises  2. Halfway home for people with SMDs |
| i16 | **Enhanced care by community health workers**  *St. John's Medical College Hospital* | Women with depression |  | 1. Screening for women with depression was done through a house-to-house survey by community health workers | 1. PHP provides the diagnosis | 1. Immediately following medical consultation, CHW visited the patients’ home to provide psychoeducation.   2. This was followed by another visit to clarify doubts and ensure medication adherence. | 1. Patients visited PHC once a month, where the physician prescribed antidepressants |  |
| i17 | **Healthier Options through Empowerment (HOPE)**  *St. John's Medical College Hospital* | People with depression or anxiety, comorbid with diabetes or cardiovascular disease |  | 1. Screening for depression, anxiety and comorbid conditions by ASHAs during community health fairs | 1. Confirmatory screening at PHC by the PHP. | 1. A group intervention called Healthy Living Group co-facilitated by a counsellor and an ASHA for problem-solving and setting short-term and long-term goals for change  2. Home visits by ASHAs to provide appointment reminders | 1. Primary care physicians at PHCs identify and treat patients   2. PHC nurses act as care managers who help with tracking patient progress   3. PHC pharmacists educate patients and their caregivers about their medication regimen, side effects and adherence.  4. Psychiatrists visit PHC to provide consultation on difficult cases | 1. Healthy Living Group builds competencies to cope with daily stressors 2. Buddy systems and peer support groups established |
| i18 | **Home Again**  *The Banyan* | People with SMDs who cannot be reintegrated with their families |  |  | 1. Individuals residing in Banyan’s psychiatric facility and selected State-run facilities identified. | 1. Assessments and reviews on medical and psychosocial support provided by personal assistants from local communities | 1. In-patient care at Banyan's Emergency Care and Recovery Center | 1. Non-institutional living arrangement, with a home shared by five residents    2. Residents are supported by personal assistants to help them identify the experiences they want to have related to work and recreation   3.Facilitation of access to opportunities and resources   4. Support is provided for residents to participate in village community meetings  5. Creation of support network in the community  6. Livelihood support  7. Help in accessing social entitlements |
| i19 | **Integrating mental health in a PHC**  *Karuna Trust (in collaboration with Government of Karnataka)* | People with SMDs | 1. Sensitization programs on mental health with village heads, Self-Help Groups and traditional healers | 1. Screening questionnaire to identify PwMI administered through focus groups with village heads and SHGs  2. Traditional healers identify and refer PwMI (due to the program's work on awareness building with them) | 1. Psychiatrist or PHPs provide diagnosis | 1. In case patients cannot visit PHCs, the clinic team led by primary care doctor (and sometimes including psychiatrist) makes home visits to provide treatment    2. Volunteers in some villages support families caring for people with SMDs and identify issues such as discontinuation of medication which they discuss with social worker   3. Community health workers and ANMs visit the families of patients who do not come for monthly follow up to the clinic, and report their progress to the doctor | 1. Integrating mental health into primary care at PHCs through a monthly mental health outpatient clinic- initial involvement of psychiatrist for diagnosis and treatment   2. Medication initially provided at cost price, and then, later, free-of-cost   3. In-patient care provided by sister organization that runs a hospital | 1. Advocacy for employment of PwMI in villages   2. Linkage to social welfare schemes   3. Support groups for PwMI and their families |
| i20 | **Integration of mental health into Self-Help Groups**  *Sampark* | Self-Help Group members (women) |  | 1. Health screening camp held at the village level to identify mental illness and other health issues |  | 1. Mental health sessions conducted during Self-Help Group meetings, focused on psychoeducation, sharing problems and building coping strategies. Initially, facilitated by NGO staff (lay health counsellors).  Following this, two SHG members trained and supervised to take the mental health sessions forward. |  |  |
| i21 | **Janamanas**  *Anjali- Mental Health Rights Organization* | People with mental distress and illness, local community members | 1. Mental health sensitization camps at street corners, local clubs and schools conducted by camp organisers from the community   2. Home visits to spread awareness about mental health and available services- conducted by outreach workers | 1. Outreach workers identify potential clients |  | 1. Follow-up home visits conducted by outreach workers  2. Mental health kiosks where counselling is provided by trained women from the community  3. Home-based counselling provided if clients cannot travel to mental health kiosk | 1. For psychiatric treatment, access to government mental hospital is facilitated | 1. Facilitating disability certification   2. Connecting with partner NGO for legal aid in case of domestic violence |
| i22 | **Maanasi**  *St. John's Academy of Health Sciences in collaboration with Rotary Bangalore Midtown and Rotary Howard West* | PwMI | 1. Mental health awareness programs conducted by community health workers in local schools, during village festivals and monthly meetings of women's cooperatives  2. Street plays to alleviate stigma and address common myths about medication | 1. Community health workers screened and identified psychiatric disorders | 1. PHP provides diagnosis | 1. Women's education and personal therapy groups conducted by social workers | 1. Psychiatric care integrated with primary care provided at an existing health center, where primary care medical officers provides diagnosis and treatment   2. Medication provided for CMDs and psychoses  3. Services - either free-of-charge or provided for a nominal fee  4. Additional health services simultaneously addressed at the clinic  5. Tertiary care at St. John's Hospital in Bengaluru | 1. Community health workers helped with job training and finding work at local day-care centers, schools and nurseries |
| i23 | **MANAS**  *Sangath* | People with Common Mental Disorders (CMDs) |  |  | 1. Screening through a tool (GHQ-12) done at a primary health facility by a Health Assistant, lay health counsellor or primary physician  2. PHP or Psychiatrist (during the visit) provides diagnosis |  | 1. Psychoeducation for all patients delivered by lay health counsellor  2. Primary health physicians at the health facility prescribed medication for people with moderate or severe CMDs   3. Interpersonal psychotherapy for people with moderate or severe CMDs, delivered by lay health counsellor   4. Psychiatrist visited the facility once a month and was available over phone | 1. Lay health counsellors provided information about social welfare services when required |
| i24 | **Mental Health Care and Research Foundation (MEHAC)**  *Mental Health Care and Research Foundation (MEHAC)* | PwMI, local community members, school children | 1. Regular community awareness programs around depression, suicide, mental health and stigma  2. Mental health literacy programs in schools | 1. Community volunteers and ASHAs identify people with mental health conditions | 1. Psychiatrist provides diagnosis | 1. Community volunteers provide follow-up care, through which they ensure medication adherence, keep track of side effects, manage emergencies and provide psychoeducation to family members | 1. Regular clinics conducted at places like PHCs, destitute homes, partner NGO office spaces, and houses of volunteers  2. Through these clinics, medication, psychotherapy and counselling provided by psychiatrists, psychologists and social workers. All services are free-of-charge. | 1. Some of the units/centers where MEHAC worked have daycare support where PwMI get skill training, supported education and employment |
| i25 | **Mental Illness Treatment Alliance (MITA)**  *the action north-east trust (the ant)* | PwMI and epilepsy |  | 1. A team of doctors including the psychiatrist provides diagnosis at camps | | 1. Regular mental health camps where patients with mental illness are provided medicines as prescribed by a team of doctors (including psychiatrist)   2. Community volunteers take history through a mobile app   3. Counsellor and social workers are available to provide services such as psychoeducation  4. Medicines dispensed by community volunteers, who provide instructions related to medicine adherence   5. All services given at low cost (INR 300) | |  |
| i26 | **Multi-pronged psychosocial intervention for people with mental health and epilepsy problems**  *Burans* | PwMI and epilepsy |  | 1. Community leaders and ASHAs selected the people receiving the intervention |  | 1. Fortnightly home visits by community mental health workers provide psychoeducation through dialogue, active listening, motivational problem solving, behavioral activation  2. Psychosocial support groups using story-based resource for critical reflection |  | 1. Financial inclusion opportunities through promoting budgeting and saving initiatives, with some groups opting to form SHGs    2. Education on government entitlements and Right to Information Act |
| i27 | **Nae Disha**  *Burans* | Youth facing distress or mental illness | 1. Peer facilitators delivered mental health promotion curriculum to young people, to build emotional resilience, reduce psychological distress, promote wellbeing and positive gender attitudes | 1. Door-to-door recruitment by peer facilitators and other team members |  | 1. Guidance to access mental health services, if required |  | 1. Peer support, discussion on legal and community support structures for gender-based violence |
| i28 | **NALAM**  *The Banyan* | PwMI, people from most marginalised backgrounds in distress | 1. Brief, group-based health promotion   2. Awareness programs | 1. Screening and referral to NALAM services by NALAM mobilisers | 1. Diagnosis done during outpatient or inpatient care. | 1. Supportive counselling   2. Home-based follow-up care by NALAM mobilisers | 1. Outpatient care with multidisciplinary teams at PHCs or community centers   2. In-patient clinical care, if required | 1. Facilitation of social entitlements by NALAM mobilisers   2. Work on land rights   3. Livelihood facilitation e.g. vocational training employment placements, referrals to training and skills development programs, interest-free loans for self-employment, livelihood aids such as tailoring machines, livestock, etc  4. Peer support groups for PwMI and their caregivers |
| i29 | **Naya Daur**  *Iswar Sankalpa* | Homeless PwMI | 1. Community awareness camps at local clubs, schools and health facilities to sensitize community members   2. Interaction with police and municipal authorities | 1. Social workers identify homeless PwMI based on observation and discussion with local community members   2. This is followed by an assessment and confirmation of diagnosis by a psychiatrist at the place where the homeless person lives. | | 1. Regular visits to street corners where homeless people live, where psychiatrist prescribes medicines and- provides psychoeducation and support | | 1. Supporting access to social entitlements by outreach social worker   2. Social worker provide clothes, if required   3. Community caregivers mobilized- they voluntarily provide basic needs like care, meals etc to the person   4. Regular community awareness camps to mobilize community caregivers and create a supportive environment for employment and reintegration  5. Providing support to access employment opportunities |
| i30 | **Participatory Learning and Action (PLA)**  *Ekjut* | Women at risk of perinatal depression, women facing violence, PwMI and their caregivers, local community members | 1. Participatory meetings for mental health awareness, to promote positive mental health amongst community members- use of picture cards, games, role-play and storytelling during these meetings, which are facilitated by peer facilitators | 1. PwMI identified by peer facilitators during community participatory meetings for mental health awareness    2. Diagnosis done as a part of collaborative telepsychiatry | | 1. Doorstep counselling services by peer facilitators  2. Collaborative telepsychiatry for PwMI at Ekjut office | | 1. Support groups for PwMI and their caregivers, conducted through Participatory Learning and Action by peer facilitators  2. Social contact program. through which PwMI share their lived experiences with the larger community to facilitate inclusion   3. Providing materials to support agriculture and poultry-related livelihood activities |
| i31 | **Programme for Improving Mental Health Care (PRIME)**  *Sangath* | People with depression, alcohol-use disorders, and psychosis | 1. Screening of a film in villages through a mobile van   2. Distribution of awareness sheets in villages | 1. Case managers identify patients in villages, based on communication with ASHAs and refer them to CHCs for further treatment | 1. Screening done at CHCs, where case managers administer screening tools for people availing general OPD or antenatal care services, and refer them to doctors, if required  2. Medical officer at CHC makes a diagnosis based on screening results | 1. Case managers provide mental health first aid to identified patients   2. Case managers conduct regular follow up visits with enrolled patients during which they also provide psychoeducation to caregivers | 1. Medical officer at CHC prescribes medication   2. Case managers provide manualised psychosocial support through Healthy Activity Program (HAP) for depression and Counselling for Alcohol Problems (CAP) for alcohol-use disorders   3. Regular visits to CHC by DMHP psychiatrist to provide consultation for severe cases |  |
| i32 | **Project Shifa**  *Padhar Hospital* | PwMI and epilepsy |  | 1. Community health workers use a screening instrument to identify people at mental disorders at the household level    2. Diagnosis by psychiatrists at camps | | 1. 1. Follow-ups by community health workers where they supervize medicines, identify crises and motivate patients to go back to meaningful work   2. One camp every 3 months, where psychiatrist evaluates patients and prescribes medication, assisted by nursing students   3. Medicines provided free-of-charge   4. Group psychoeducation sessions for people with severe mental disorders and their families 2. 5.People with Common Mental Disorders and substance-use disorders treated at Padhar Hospital | |  |
| i33 | **Psychosocial support in tsunami-affected areas**  *Schizophrenia Research Foundation (SCARF)* | People in tsunami-affected areas | 1. Screening short film on tsunami to dispel rumours | 1. Community level workers focused on identification    2. Diagnosis at outpatient services and through telepsychiatry | | 1. Community level workers provide psychosocial interventions through one-on-one interactions for emotional support, group activities such as collective grieving, dance and music   2. Camps to provide psychiatric outpatient and telepsychiatry services   3. Medicines given free-of-cost   4. Psychoeducation provided to caregivers to reduce stigma | | 1. Guidance and practical help for housing provided by community level workers |
| i34 | **SAWAB Intervention**  *Supporting Always Wholeheartedly All Broken Hearted (SAWAB) (in collaboration with an academic institution)* | Youth with mental disorders in a conflict-prone region | 1. Awareness workshops and meetings with faith healers, schoolteachers, local government leaders and community health personnel to raise awareness about and destigmatise mental illness and to improve identification and management of mental disorders   2. Creating short programs that use drama and song to disseminate information about mental disorders- screened on local TV and radio | 1. LHWs identify patients in villages through their knowledge of the local community, conduct an informal assessment and arrange for an appointment with a psychiatrist   2. Traditional healers also identify patients | 1. Psychiatrist at clinic provides formal diagnosis | 1. Follow-up visits by LHWs along with regular phone-based support   2. Home visit by psychiatrist in case a patient is unable to visit the clinic | 1. Psychiatrist at clinic prescribes medicines and refers for counselling, if required   2. Medicines provided free-of-cost   3. Counselling provided by psychotherapist, with support from LHWs | 1. LHWs facilitate social, occupational and academic reintegration   2. LHWs work with patients' families, teachers and peers to reduce stigma |
| i35 | **SCARF Telepsychiatry in Puddukottai (STEP)**  *Schizophrenia Research Foundation (SCARF)* | People with SMDs | 1. Screening of short films on mental health on a mobile bus  2. Street plays to sensitize community   3. Sensitization for local NGOs, social workers, and police for referral  4. Mental health and suicide prevention committees a village level | 1. Community level workers screen and identify PwMI and refer them to the clinic    2. Diagnosis through teleconsultation through bus that travels close to patients | | 1. Teleconsultation provided in a bus that travels close to patients’ homes: a psychiatrist from Chennai connected with patients and caregivers and prescribes medicines   2. Medication given free-of-cost from a pharmacy that is on the bus   3. Follow-up home visits conducted by community level workers to ensure treatment compliance and give psychoeducation to families   4. Mobile app used for tracking and sending reminders to patients on appointments and taking medication | | 1. Community level workers facilitate disability benefits and connections with social schemes  2. Community level workers liaised with local NGOs and government schemes to get jobs for patients   3. Self-Help Groups of service users and family members created |
| i36 | **Seher Urban Community Mental Health and Inclusion Programme**  *Bapu Trust* | People with mental distress, illness, and psychosocial disability in a low-income slum community | 1. Community awareness meetings | 1. Community-based household surveys and field workers' observations used to identify people in need of services   2. Assessment of psychosocial, economic and family needs of a client and referrals made based on this-- assessment team may comprise of project leader, counsellor, arts-based therapist, senior field counsellors and grassroots peer supporters. |  | 1. Support plan made based on assessment-  client offered a choice of solutions under various headers like self-care, nutrition, individual and group support. comprehensive healthcare, addressing social justice issues like violence    2. A basket of home-based psychosocial services provided by non-formal caregivers   3. Therapeutic groups using modalities like peer support, arts-based therapy and talk therapy in the community- anchored by facilitators and field workers from the community | 1. Active partnerships with general practitioners, psychiatrists, AYUSH practitioners | 1. Mobilizing support systems (including family members, neighbors and community actors) for the client using the framework of a circle of care: Safety circle who the client can access in times of crisis   2. Working with family members on caregiving and support skills, ensuring responsible and accountable caregiving without violence or seclusion |
| i37 | **SHIFA**  *Emmanuel Hospital Association* | People with mental distress and illness, general community members | 1. Large group awareness programs on mental health   2. Small group meetings to build skills and capacities around mental health   3. Workshops with traditional healers, religious leaders and local doctors on active listening and referral of people with severe mental distress to biomedical care | 1. PwMI and distress identified by community volunteers and program staff | 1. Diagnosis at primary mental health clinics | 1. Community volunteers and SHIFA staff provided social and psychological support | 1. Primary mental health care clinic held fortnightly at a government health center providing both biomedical and non-pharmacological therapy | 1. Facilitating access to government entitlements and disability certificates   2. Strengthening community voice to advocate for care and support through the formation of community-based disabled persons groups (DPGs) |
| i38 | **Systematic Medical Appraisal, Referral and Treatment (SMART)**  *The George Institute for Global Health* | People with CMDs | 1. Stigma reduction campaign through door-to-door visits, showing videos on PwMI talking about their experience, distribution of brochures, putting up posters, and theatre performance by a local group | 1. ASHAs screen people for CMDs during routine visits to community, through Electronic Decision Support System (EDSS) app    2. Primary care doctors diagnose patients at PHCs and health camps | | 1. Primary care doctors provide treatment, either at health camps in villages or at PHCs  2. Follow-up by ASHAs, with follow-up plan being provided through EDSS on their phones | |  |
| i39 | **Thinking Healthy Programme (THP)**  *Sangath* | People with perinatal depression |  |  | 1. Participants recruited from PHCs or antenatal clinics based on their PHQ-9 scores | 1. Peer delivery of behavioral activation sessions at participant's home or other venue chosen by participant |  |  |
| i40 | **Vidarbha Stress and Health Program (VISHRAM)**  *Sangath* | People at risk of suicide due to depression and alcohol-use disorders | 1. Awareness meetings conducted by community health workers   with small groups of 12-15 people, invited through SHG and farmer's group meetings  2. Household visits to increase awareness of mental health conditions and inform people about services   3. Screening of a film using clips from popular cinema to spread awareness of mental disorders | 1. Community health workers identify people with depression, provide psychological first aid and referral | 1. Physicians at PHCs provide diagnosis | 1. Lay counsellors provide Health Activity Program (a brief structured psychosocial program for depression) at the village level | 1. Lay counsellors provide Health Activity Program (a brief structured psychosocial program for depression) at health facilities including PHCs   2. Physicians at PHCs prescribe medicines, if required   3. Outpatient clinics at rural hospitals conducted by psychiatrists |  |
| i41 | **Yuva Spandana**  *Department of Youth Empowerment and Sports, Government of Karnataka* | Youth | 1. Youth mental health promotion through guidance centers established in each district, where youth volunteers provide guidance on topics such as health and lifestyle, education, academic and career issues, relationship issues, personality development and emotional issues, gender, sex and sexuality and safety issues.   2. Community sensitization programs with parents and caregivers on issues faced by youth |  |  |  |  |  |
